# Impact of parathyroid gland classification on hypoparathyroidism following total thyroidectomy with central neck dissection for differentiated thyroid cancer

**DOI:** 10.1080/07853890.2025.2476223

**Published:** 2025-03-11

**Authors:** Qixuan Sheng, Wei Li, Ping Zhang, Qiang Wang, Siluo Zha, Wensheng Rao, Bin Wang, Xinyun Xu, Ming Qiu, Wei Zhang, Chengxiang Shan

**Affiliations:** Department of Thyroid, Breast and Hernia Surgery of Changzheng Hospital affiliated to Naval Military Medical University, Shanghai, China

**Keywords:** Hypoparathyroidism, parathyroid glands, classification, differentiated thyroid cancer

## Abstract

**Objective:**

To assess the impact of parathyroid gland (PG) classification on hypoparathyroidism incidence following total thyroidectomy (TT) with central neck dissection (CND) in patients with differentiated thyroid carcinoma (DTC).

**Methods:**

In this prospective cohort study, adult patients with DTC who underwent TT with CND between 2021 and 2023 were enrolled, with a maximum follow-up duration of 32 months. A simplified PG classification system was employed, categorizing glands into four distinct types: tightly connected, loosely connected, non-connected, and thymic. The intraoperative frequency of each PG type was recorded based on this classification. Parathyroid hormone (PTH) levels were routinely tested 1 day, 1 month, 6 months and 1 year after surgery. The association between PG classification and the incidence of postoperative hypoparathyroidism was then systematically analysed.

**Results:**

Among 135 patients with DTC (mean age: 48.50 ± 10.52 years; 101 women), 62 patients (45.93%) developed hypoparathyroidism on postoperative day 1 (POD1), while 14 patients (10.37%) experienced hypoparathyroidism on postoperative month 1 (POM1). All patients exhibited PTH normalization within six months, with no permanent hypoparathyroidism cases. A total of 532 PGs were identified: 264 (49.62%) were tightly connected, 150 (28.20%) loosely connected, 95 (17.86%) non-connected, and 23 (4.32%) thymic. The highest prevalence of hypoparathyroidism on POD1 was observed in patients with four tightly connected PGs (*p* < 0.001). Patients with four tightly connected PGs had a significantly greater incidence of hypoparathyroidism than those with none (*p* = 0.024). Regression analysis revealed that each additional tightly connected PG increased the risk of hypoparathyroidism by 1.38 times (*p* = 0.019). Tightly connected PGs demonstrated predictive value for POD1 hypoparathyroidism (AUC = 0.604, cut-off: two tightly connected glands). In contrast, thymic PGs did not provide a protective effect.

**Conclusion:**

PG classification may serve as a valuable tool for surgeons in intraoperative parathyroid preservation and the prediction of postoperative hypoparathyroidism in patients with DTC. Notably, DTC patients with more than two tightly connected PGs are at an elevated risk of developing temporary hypoparathyroidism, emphasizing the importance of meticulous parathyroid preservation during surgical procedures.

## Introduction

Hypoparathyroidism is widely acknowledged as the most common complication following total thyroidectomy (TT) combined with central neck dissection (CND) [1,2]^.^ A retrospective analysis of patients with differentiated thyroid carcinoma (DTC) reported an incidence of hypoparathyroidism on postoperative day 1 (POD1) of 48.69% after TT with CND, with severe hypoparathyroidism observed in 17% of cases [3]^.^ This condition poses significant symptomatic challenges and carries potentially life-threatening risks. Moreover, hypoparathyroidism contributes to extended hospital stays, necessitates replacement therapy, and requires prolonged post-discharge monitoring, all of which negatively impact patients’ overall quality of life [4,5].

When TT and CND are performed concurrently, preserving the integrity and functionality of the parathyroid glands (PGs) poses a significant challenge [6]. This difficulty arises due to the anatomical variability of the PGs and the susceptibility of their vascular supply to damage during surgical procedures [2]. To mitigate this risk, meticulous capsular dissection techniques are commonly utilized [7], avoiding ligation of the main branches of the superior and inferior thyroid arteries to protect the terminal blood vessels supplying the PGs [8]. Furthermore, advancements in PG tracking technologies, such as carbon nanoparticles [9], indocyanine green (ICG) angiography [10,11] and near-infrared autofluorescence (NIRAF) [12], have demonstrated significant efficacy in enhancing the accurate identification of PGs. These technologies play a critical role in the preservation of parathyroid function during surgery.

Numerous studies have underscored the pivotal role of PG classification in determining the incidence of hypoparathyroidism following thyroid surgery [13–16]. For example, PGs classified as “tightly connected types” are closely adherent to the thyroid gland and lack a singular nutrient artery; instead, their blood supply is derived from fragile terminal branches traversing the thyroid parenchyma [17,18]. This anatomical feature renders these glands particularly vulnerable to ischemia when separated from the thyroid capsule during surgery. Conversely, ‘thymic-type’ PGs, located within the thymus, are generally less susceptible to surgical damage. Theoretically, an increased number of tightly connected PGs is inversely correlated with the presence of thymic PGs [19], potentially elevating the risk of postoperative hypoparathyroidism. However, this hypothesis has yet to be extensively validated through prospective studies. Moreover, considering that humans typically possess four PGs, dysfunction of a single gland may not significantly impair overall parathyroid function, thereby potentially mitigating the risk of hypoparathyroidism [20]. The threshold number of tightly connected PGs required to precipitate hypoparathyroidism, as well as the compensatory effect of thymic PGs, remains uncertain and warrants further investigation.

Building upon existing scholarly classifications [14,15,17] and informed by our own clinical observations, a simplified PG classification system was developed and implemented in a prospective clinical trial. This classification system was utilized to systematically identify each PG in patients with DTC undergoing TT with CND. PTH levels were routinely tested as a measure of parathyroid function. The primary aim of this study was to evaluate the impact of the PG classification system on the incidence of postoperative hypoparathyroidism.

## Methods

### Study design

Between April 2021 and December 2023, consecutive DTC patients who underwent TT along with CND at the Department of Thyroid, Breast and Hernia Surgery of Changzheng Hospital were enrolled in this prospective study. The inclusion criteria for the study were: (1) Patients with DTC, aged 18–70 years, of either sex; (2) requirement for TT with CND. The exclusion criteria included: (1) lobectomy or isthmectomy only; (2) intraoperative parathyroid auto-transplantation; (3) simultaneous lymph node dissection in the lateral cervical region; and (4) preexisting parathyroid diseases.

The trial was registered in the China Clinical Trial Registry (ChiCTR2000039788) on November 10, 2020. Ethical approval was obtained from the Ethics Committee of Changzheng Hospital affiliated to Naval Military Medical University(2021SL009). All patients entered the study with full knowledge and written informed consent. The study strictly adhered to the principles of the Declaration of Helsinki.

### Surgical procedures

All patients underwent standard total thyroidectomy, ensuring complete removal of thyroid tissue with no residual remnants. The PGs were carefully identified and documented using the naked eye, then classified into specific categories. All PGs were preserved and left *in situ* without auto-transplantation. The TT and CND procedures were summarized as follows: (1) A precise 5 cm curved cervical incision was carefully created above the suprasternal notch. The dissection was conducted along the sub-platysmal plane, followed by a longitudinal incision through the linea alba cervicalis. (2) Exposed and centrally divided the thyroid isthmus. (3) Mobilized the pyramidal lobe and Delphian lymph nodes. (4) Fully exposed the lateral and inferior poles of the thyroid. (5) Identified inferior parathyroid gland (IPG) at the lower thyroid pole and classified the IPG. If not found, the IPG might probably be in the central neck compartment. (6) Exposed and dissected the superior pole of the thyroid. (7) Identified and classified superior parathyroid gland (SPG). (8) Exposed recurrent laryngeal nerve and removed thyroid lobe entirety along the surface of nerve. (9) Performed CND. Identified and classified IPG in the central neck compartment. If the IPG was identified as thymic type, meticulously preserved the thymus tongue. (10) If an IPG was not found either at the lower thyroid pole or around the thymus, carefully inspect the region of the thyrothymic ligament to identify the IPG and preserve its pedicle.

### Classifications of PGs

Based on the location of the PGs, their relationship with the thyroid and thymus, and their blood supply, combined with previously reported PG classifications and our clinical experience, we have categorized the PGs into four types (Table 1).

**Table 1.** The details of the new classification of parathyroid glands.

Type	Description	Location	Difficulty of preservation
I	Tightly connected	Tightly adheres to thyroid gland (co-capsular)	Difficult
II	Loosely connected	Loosely adheres to thyroid gland (co-capsular)	Challenging
III	Non-connected	Lacks direct anatomical connections with the thyroid gland	Moderate
IV	Thymic	nestled within the thymus	Simple

**Figure 1.**
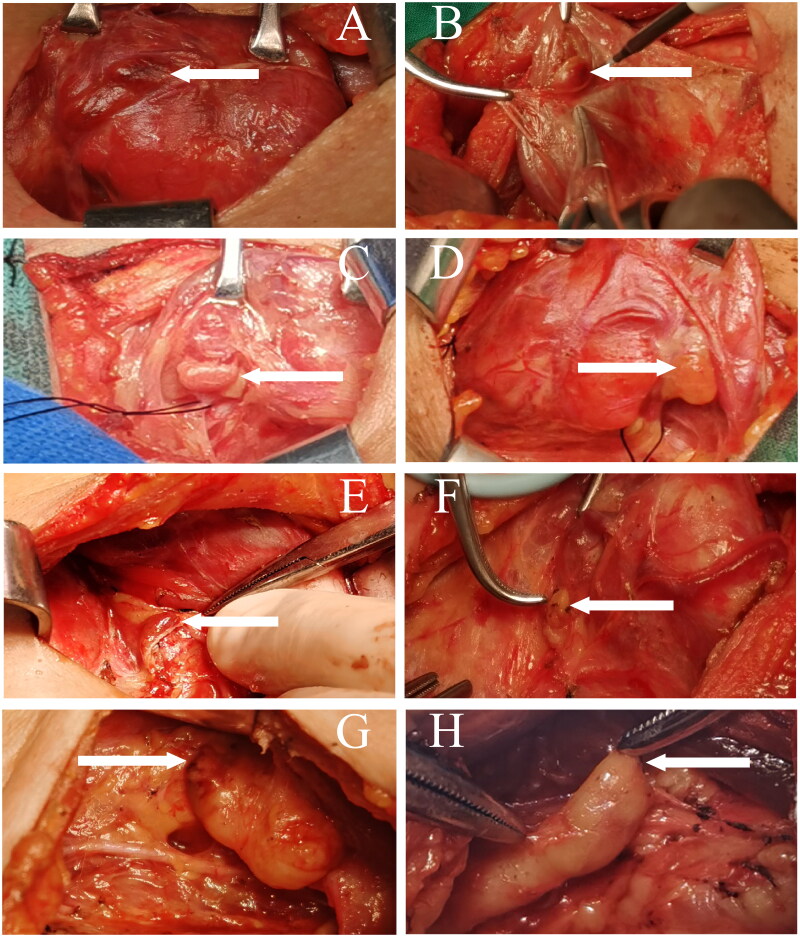
Intraoperative figures description of types of parathyroid gland. (A-B) Type I PG is firmly adherent to the thyroid gland and located within the thyroid capsule. (C-D) Type II PG shares a common capsule with the thyroid gland (co-capsular), though the connection between them is relatively loose. (E-F) Type III PG is typically located around the thyroid-thymus ligament and adjacent to arterial sheaths. (G-H) Type IV PG is embedded within the thymus, forming a unified structure with it.

The type I PG, also known as the tightly connected PG, is firmly adherent to the thyroid gland and located within the thyroid capsule (co-capsular). It is primarily supplied by fine terminal branches that traverse the thyroid parenchyma. During thyroidectomy, this type of PG can be preserved *in situ*, remaining connected to the surrounding tissue by minimal fibrous tissue. (Figure 1(A–B)).

The type II PG, also known as the loosely connected PG, shares a common capsule with the thyroid gland (co-capsular), though the connection between them is relatively loose. The blood supply to this type of PG does not primarily depend on the thyroid. After dissection, the PG is typically left *in situ*, connected to the surrounding tissue by a greater amount of fibrous tissue. (Figure 1(C–D)).

The type III PG, also known as the non-connected PG, lacks direct anatomical connections with the thyroid gland and does not share a common capsule. It is commonly supplied by a branch of the inferior thyroid artery. During surgery, it is often separated from the thyroid by fat or fibrous connective tissue. (Figure 1(E–F)).

The type IV PG, also known as the thymic PG. It is embedded within the thymus, forming a unified structure with it. Upon opening the thymus, the PG becomes visible. (Figure 1(G–H)).

### Outcomes

PTH was routinely tested 1 day, 1 month, 6 months and 1 year after surgery. Hypoparathyroidism was defined as a PTH level < 15 pg/mL, categorized as mild (10 pg/mL ≤ PTH < 15 pg/mL), moderate (5 pg/mL ≤ PTH < 10 pg/mL), and severe (PTH < 5 pg/mL). Transient hypoparathyroidism was PTH < 15 pg/mL within the first 6 months, while permanent hypoparathyroidism was PTH < 15 pg/mL beyond 6 months.

### Perioperative management

All patients underwent standard perioperative management. No preventive calcium supplementation was given. Oral calcium and vitamin D supplementation(600 mg of calcium and 125 IU of vitamin D3, 2 tablets taken three times daily) was conducted to the patients with a PTH level < 15 pg/mL or serum calcium level <2.18 ± 0.09 mmol/L on POD 1, and intravenous calcium supplementation (3 g of calcium gluconate, administered twice daily for at least 3 days) was added to those with a PTH level < 5 pg/mL or severe symptomatic hypocalcemia. The duration of initial oral calcium and vitamin D supplementation was 1 month and adjustments were made dynamically until the patients had normal serum calcium levels.

### Statistical analysis

Statistical analysis was performed using SPSS version 26.0 software. Continuous variables meeting normality and homoscedasticity criteria were analysed by independent-samples T test, expressed as mean ± standard deviation. Categorical data were tested using the Chi-square test or Fisher tests, reported with counts, percentages, and 95% CIs. Factors with *p* < 0.05 in univariate analysis were included in logistic regression to build a predictive model for postoperative hypoparathyroidism. *p* < 0.05 was considered statistically significant.

## Results

### Baseline characteristics of patients

Between April 2021 and December 2023, a total of 135 adult patients with DTC who underwent TT with CND were analyzed. The study cohort included 101 women (74.81%) and 34 men (25.19%), with a mean age of 48.50 ± 10.52 years (range, 24–69). The average height was 163.32 ± 7.37 cm, the average weight was 64.27 ± 12.09 kg, and the average BMI was 23.06 ± 5.89. Among the 135 DTC patients, 43(31.85%) had hypertension, 9 (6.67%) had diabetes, and 56 (41.48%) had Hashimoto’s disease as comorbidities. The preoperative PTH level was 49.67 ± 16.8 pg/mL, the TSH level was 2.31 ± 1.84 mIU/L, the serum calcium level was 2.18 ± 0.09 mmol/L, the serum phosphorus level was 1.23 ± 0.18 mmol/L, and the vitamin D level was 24.24 ± 8.93 ng/mL. No losses occurred during the follow-up period, which ranged from 1 month to 32 months.

### Incidence of hypoparathyroidism

On POD1, 62 out of 135 DTC patients (45.93%) developed hypoparathyroidism, with an average PTH level of 20.86 ± 16.05 pg/mL, an average serum calcium level of 1.99 ± 0.15 mmol/L, and an average serum phosphorus level of 1.5 ± 0.31 mmol/L. On postoperative month 1 (POM1),14 patients (10.37%) still had hypoparathyroidism, with an average PTH level of 33.96 ± 16.03 pg/mL, an average serum calcium level of 2.18 ± 0.15 mmol/L, and an average serum phosphorus level of 1.18 ± 0.18 mmol/L. By 6 months postoperatively, PTH levels had normalized in all patients, with no instances of permanent hypoparathyroidism observed. On POD1, the incidence of mild, moderate, and severe hypoparathyroidism was 10.37%, 25.93%, and 9.63%, respectively. On POM1, the incidence of mild, moderate, and severe hypoparathyroidism had decreased to 2.96%, 5.19%, and 2.22%, respectively.

### Identification and classification of PGs

**Figure 2.**
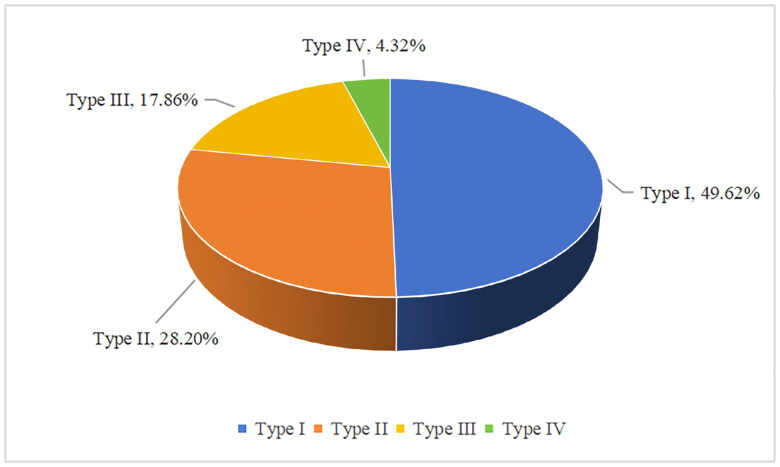
The proportional composition of each parathyroid gland type.

In a cohort of 135 patients, a total of 532 PGs were identified, comprising 264 Type I glands (49.62%), 150 Type II glands (28.2%), 95 Type III glands (17.86%), and 23 Type IV glands (4.32%), with Type I being the most prevalent (Figure 2).

**Figure 3.**
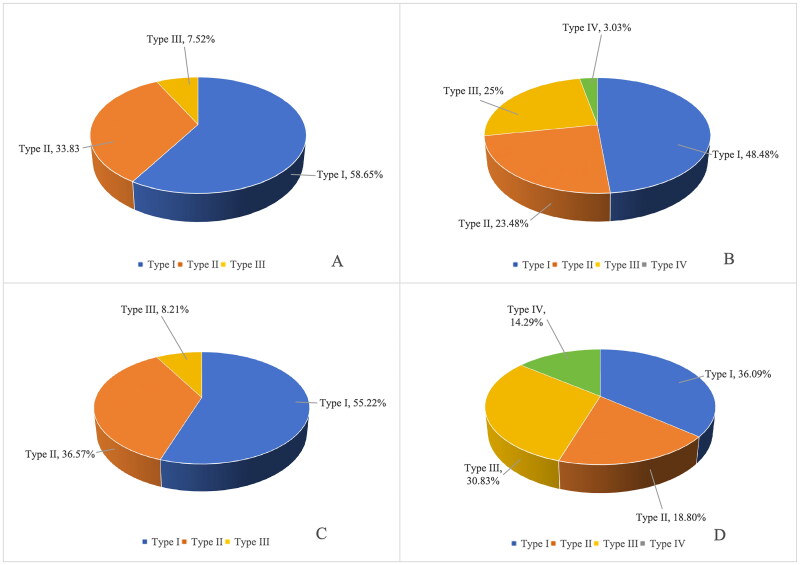
Identification of parathyroid glands. (A)Proportional composition of the right superior parathyroid gland. (B) Proportional composition of the right inferior parathyroid gland. (C) Proportional composition of the left superior parathyroid gland. (D)Proportional composition of the left inferior parathyroid gland.

Among the 133 right superior PGs, 78 were classified as Type I (58.65%), 45 as Type II (33.83%), and 10 as Type III (7.52%), with no Type IV glands detected (Figure 3(A)). In the right inferior position, 132 PGs were identified, including 64 Type I (48.48%), 31 Type II (23.48%), 33 Type III (25%), and 4 Type IV glands (3.03%) (Figure 3(B)). For the left superior PGs, 134 were identified, with 74 classified as Type I (55.22%), 49 as Type II (36.57%), and 11 as Type III (8.21%), with no Type IV glands observed (Figure 3(C)). Finally, among the 133 left inferior PGs, 48 were Type I (36.09%), 25 were Type II (18.8%), 41 were Type III (30.83%), and 19 were Type IV (14.29%) (Figure 3(D)) (Table 2).

**Table 2.** The identification and classification of parathyroid glands.

PG location	Type I	Type II	Type III	Type IV
Right Superior	78(58.65)	45(33.83)	10(7.52)	0(0)
Right Inferior	64(48.48)	31(23.48)	33(25)	4(3.03)
Left Superior	74(55.22)	49(36.57)	11(8.21)	0(0)
Left Inferior	48(36.09)	25(18.8)	41(30.83)	19(14.29)

Abbreviation: PG, parathyroid gland.

### Impact of PGs on hypoparathyroidism

On POD1, among the 62 patients who developed hypoparathyroidism, the group with four tightly connected PGs had the highest number of individuals (15 cases), followed by the group with two tightly connected and two loosely connected PGs (8 cases), with a statistically significant difference in distribution (Chi-square value = 76.81, *p* < 0.001). When grouping patients with two or more tightly connected PGs together and those with fewer than two tightly connected PGs into another group, the former had significantly more cases of hypoparathyroidism than the latter (44 vs. 18), also with a statistically significant difference (Chi-square value = 10.90, *p* < 0.001). However, no specific combination of PG types was found to be more prone to developing hypoparathyroidism on POM1.

**Figure 4.**
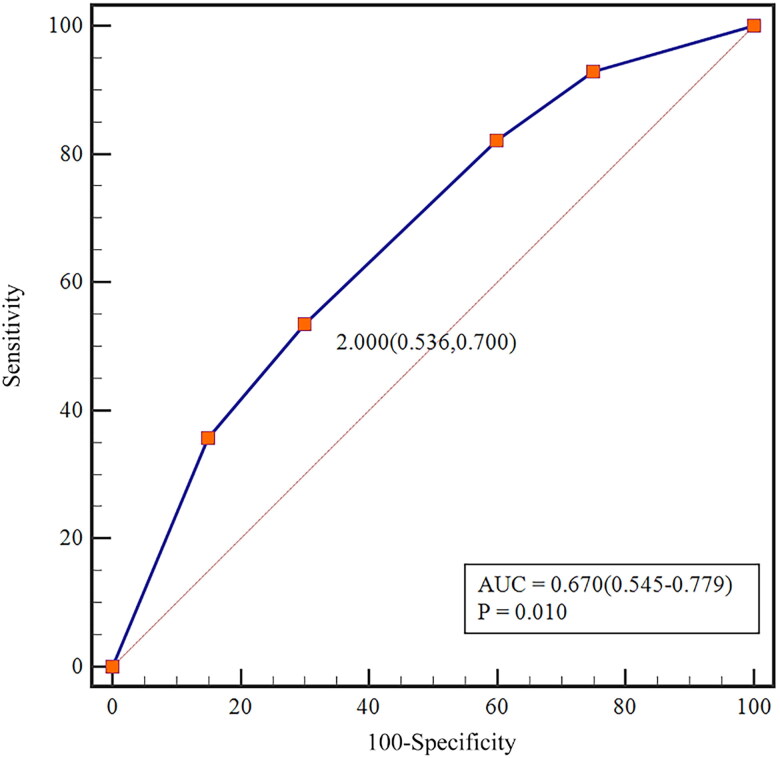
ROC Curve analysis of independent predictive factors for POD1 hypoparathyroidism. It shows an AUC of 0.604, sensitivity of 0.403, specificity of 0.740, and a cut-off value of 2.

Based on the number of thymic PGs, patients were divided into two subgroups: those without a thymic PG and those with 1 or 2 thymic PGs. Based on the number of tightly connected PGs, patients were stratified into five subgroups with counts ranging from 0 to 4. Among all 135 patients, whether on POD1 or POM1, the incidence of hypoparathyroidism was notably higher in patients without a thymic PG compared to those with 1 or 2. However, this difference did not reach statistical significance (Table 3). Additionally, no significant difference in the incidence of hypoparathyroidism was observed between groups when considering the number of tightly connected PGs (Table 4). However, when treating tightly connected PGs as a categorical variable in regression analysis, we found that each additional tightly connected PG was associated with a 1.38-fold increase in the incidence of hypoparathyroidism (*p* = 0.019). Furthermore, the incidence of hypoparathyroidism was significantly higher in patients with four tightly connected PGs compared to those without any, with statistical significance (*p* = 0.024) (Table 5). Receiver operating characteristic (ROC) curve analysis demonstrated that tightly connected PGs had a moderate predictive effect on the occurrence of POD1 hypoparathyroidism, with an AUC of 0.604, sensitivity of 0.403, specificity of 0.740, and a cut-off value of 2 (Figure 4). However, regression analysis did not confirm thymic PGs as a protective factor against hypoparathyroidism.

**Table 3.** The POD1 hypoparathyroidism and POM1 hypoparathyroidism in the 2 groups.

Thymic PG(No.)	POD1 Hypoparathyroidism			POM1 Hypoparathyroidism		
	YES	NO	*P* value	YES	NO	*P* value
0	56(90.32)	58(79.45)	0.082	14(100)	100(82.64)	0.191
1-2	6(9.68)	15(20.55)		0(0)	21(17.36)	

Abbreviation: PG, parathyroid gland. POD1,postoperative day 1. POM1,postoperative month 1.

**Table 4.** The POD1 hypoparathyroidism and POM1 hypoparathyroidism in the 5 groups.

Tightly connected PG(No.)	POD1 Hypoparathyroidism			POM1 Hypoparathyroidism		
	YES	NO	P value	YES	NO	*P* value
0	8(12.9)	17(23.29)	0.295	2(14.29)	23(19.01)	0.754
1	10(16.13)	14(19.18)		4(28.57)	20(16.53)	
2	19(30.65)	23(31.51)		3(21.43)	39(32.23)	
3	10(16.13)	10(13.7)		2(14.29)	18(14.88)	
4	15(24.19)	9(12.33)		3(21.43)	21(17.36)	

Abbreviation: PG, parathyroid gland. POD1,postoperative day 1. POM1,postoperative month 1.

**Table 5.** Univariate and multivariate analysis for factors associated with POD1 hypoparathyroidism.

Factor	Univariate Analysis		Multivariate Analysis	
	OR(95%CI)	P value	OR(95%CI)	P value
**Tightly connected PG**	1.33(1.03–1.73)	0.032	1.38(1.05–1.81)	0.019
No.1 VS No.0	1.52(0.47–4.88)	0.484		
No.2 VS No.0	1.76(0.62–4.95)	0.288		
No.3 VS No.0	2.13(0.63–7.16)	0.224		
No.4 VS No.0	3.54(1.09–11.51)	0.035	4(1.2-13.33)	0.024
**Thymic PG**	0.41(0.16–1.06)	0.066		
No.1-2 VS No.0	0.41(0.15–1.14)	0.089		

Abbreviation: PG: parathyroid gland; POD1: postoperative day 1; OR: odds ratio.

## Discussion

Previous studies have explored the influence of PG classification on the decline in parathyroid function after thyroid surgery [14,15,17]. Our research, however, focused on a defined cohort of patients with DTC undergoing TT with CND. Unlike earlier studies that recommended the transplantation of PGs with compromised vascular supply [21,22], we prioritized the *in situ* preservation of all PGs, irrespective of their classification. To the best of our knowledge, this is the first prospective study to systematically assess the impact of preserving all PGs in their native anatomical locations on the incidence of postoperative hypoparathyroidism.

It is reasonable to hypothesize that the extent of injury sustained by different types of PGs during thyroid surgery is influenced by their distinct anatomical and vascular characteristics [23]. Consequently, some researchers propose that variations among PG types may play a pivotal role in the development of postoperative hypoparathyroidism. Cui et al. [15] categorized PGs into five subgroups (A/B1/B2/B3/C) based on their vascular supply and anatomical relationship with the thyroid gland, recommending auto-transplantation for PGs classified as types B2 and C to preserve function. Similarly, Zhu et al. [14] classified PGs into six subgroups (A1/A2/A3/B1/B2/B3), noting that type A PGs are particularly challenging to preserve during surgery. Additionally, Hou et al. [17] focused on the inferior PGs, categorizing them into four subgroups primarily based on their vascular supply. Ji et al. introduced the Index of Parathyroid Viability Score (IPVS) as a novel quantitative metric and demonstrated a significant correlation between higher IPVS values and an increased incidence of both transient and permanent hypoparathyroidism [23]. While these studies provide detailed classifications of PGs, a significant limitation lies in the complexity of the classification systems, which include an excessive number of subtypes.

To address this, our classification adopts a simplified approach, classifying PGs into four types: Type I (tightly connected) PG, Type II (loosely connected) PG, Type III (non-connected) PG, and Type IV (thymic) PG. By training surgeons to identify and classify PGs, tailored management strategies can be applied to different types of PGs. For both tightly connected and loosely connected PGs, which are primarily vascularized by capillaries originating from the capsule and supplied by small branches of the thyroid arteries [24], it is crucial to preserve the surrounding capsule. During dissection, efforts should focus on maintaining the fibrous connective tissue around the PG, with *in situ* preservation being a reliable approach. For non-connected PGs, which receive blood supply from small branches of the superior and inferior thyroid arteries, careful dissection near the thyroid’s true capsule is necessary. The main trunk of the thyroid arteries and pedicles involving nutrient branches should be preserved to maintain blood flow to the PGs. For thymic PGs, typically supplied by branches of the internal thoracic or inferior thyroid arteries, it is crucial to protect the thymus and preserve the branches of the inferior thyroid artery directed toward it, ensuring the PGs’ vascularization. These strategies, based on PG classification, help preserve parathyroid function and minimize complications during thyroid surgery.

Based on the location of PGs, their relationship with the thyroid and thymus, and their blood supply, we designated tightly connected PG as the most susceptible to damage and thymic PG as the least susceptible to damage. This streamlined method may assist surgeons in more accurately predicting which patients are at an increased risk of postoperative hypoparathyroidism. Our findings indicate that patients with four tightly connected PGs are most at risk for hypoparathyroidism, supporting our hypothesis that the highest incidence occurs in this group [25]. However, no significant difference in hypoparathyroidism incidence was observed between groups with 1, 2, or 3 tightly connected PGs. As humans typically have four PGs, dysfunction of a single gland may not substantially impact overall parathyroid function. These results suggest that during TT with CND, the risk of hypoparathyroidism is significantly elevated only when ischemic injury affects all four PGs, consistent with previous studies [1,26,27]. Additionally, regression analysis revealed that with each additional tightly connected PG, the risk of hypoparathyroidism increased by a factor of 1.38, with a threshold at two tightly connected PGs. Therefore, patients with more than two tightly connected PGs require careful preservation during surgery to reduce hypoparathyroidism risk. Theoretically, patients with thymic PGs should be less susceptible to hypoparathyroidism, as the integrity and vascular supply of these glands are preserved during surgery. However, our study did not observe a protective effect of thymic PGs against hypoparathyroidism. We attribute this to the small sample size, as only 21 patients (15.56%) in our cohort had thymic PGs.

In our study, PG tracking technologies were not employed for localization purposes. The utilization of such methods could have potentially diminished the emphasis placed on PG classification. Existing literature highlights that technologies such as ICG angiography and NIRAF significantly enhance the efficiency and accuracy of PG localization, thereby facilitating their preservation. These approaches have been demonstrated to reduce the incidence of postoperative hypocalcaemia and preserve parathyroid function [10–12]. However, it is essential to acknowledge the limitations associated with the application of these tracking technologies. Their use necessitates specialized equipment, which is available in only a limited number of hospitals across China. Additionally, the integration of such techniques into surgical workflows may prolong operative times due to equipment adjustments, potentially disrupting the overall surgical schedule. Moreover, while tracking technologies improve the precision of PG identification [28], the pivotal factor in preserving parathyroid function remains the meticulous dissection of the glands from surrounding tissues, whether identified visually or through technological assistance. Given these considerations, our routine practice prioritizes visual identification of the PGs for their protection. We maintain that clinical expertise, developed through extensive training and experience, provides the most dependable foundation for the accurate localization and preservation of the PGs during surgery.

Parathyroid auto-transplantation is widely recognized as an effective strategy for managing PGs that become de-vascularized and cannot be preserved *in situ*, potentially mitigating the risk of permanent hypoparathyroidism [14,17,29]. However, the intraoperative evaluation of parathyroid ischemia and congestion, which often relies solely on visual evaluation of gland color, is inherently subjective and prone to variability [30]. The potential for parathyroid tissues, even those connected by minimal fibrous tissue, to re-establish vascular supply and recover functionality remains insufficiently understood. In contrast, our study employed *in situ* preservation for all PGs. Although some glands connected by minimal fibrous tissue exhibited signs of ischemia or congestion during surgery, long-term postoperative PTH levels did not reveal an increased incidence of permanent hypoparathyroidism. These findings suggest that *in situ* preservation is a reliable approach for maintaining the function of both tightly connected and loosely connected PGs.

### Future perspective

Our statistical analysis reveals that the presence of more than two tightly connected PGs is a significant predictor of POD1 hypoparathyroidism. Therefore, the need for proactive auto-transplantation of PGs, particularly when two or more are tightly connected, warrants further investigation. This research will help ascertain whether such an intervention could effectively reduce the incidence of temporary postoperative hypoparathyroidism.

### Limitations

The findings of this study have to be seen in the light of some limitations. This is a single-center study includes 135 patients; therefore, future studies will validate the findings on samples or shared data from multiple centers to cover the diversity of the samples. Also, our results need to be validated with larger cohorts and more surgeons to evaluate our model’s generalization. In addition, the subjectivity in PG classification, surgical complexity, and variability among surgeons may affect the outcomes; therefore, future studies will adapt a double-blind approach or expand the surgical team to reduce bias. Despite these limitations, we have demonstrated the feasibility and importance of using PG classification for intraoperative parathyroid protection and postoperative hypoparathyroidism prediction of DTC patients.

## Data Availability

Our research data contains sensitive patient information, so it is unsuitable to share directly online. Data sharing is available upon reasonable request. You may contact the corresponding author by email to obtain the original research data.
